# Coverage of routine reporting on malaria parasitological testing in Kenya, 2015–2016

**DOI:** 10.1080/16549716.2017.1413266

**Published:** 2017-12-20

**Authors:** Joseph K. Maina, Peter M. Macharia, Paul O. Ouma, Robert W. Snow, Emelda A. Okiro

**Affiliations:** ^a^ Malaria Public Health Department, Kenya Medical Research Institute-Wellcome Trust Research Programme, Nairobi, Kenya; ^b^ Centre for Tropical Medicine and Global Health, Nuffield Department of Clinical Medicine, University of Oxford, Oxford, UK

**Keywords:** Routine data, malaria risk mapping, DHIS2, geographic coverage

## Abstract

**Background:** Following the launch of District Health Information System 2 across facilities in Kenya, more health facilities are now capable of carrying out malaria parasitological testing and reporting data as part of routine health information systems, improving the potential value of routine data for accurate and timely tracking of rapidly changing disease epidemiology at fine spatial resolutions. **Objectives:** This study evaluates the current coverage and completeness of reported malaria parasitological testing data in DHIS2 specifically looking at patterns in geographic coverage of public health facilities in Kenya. **Methods:** Monthly facility level data on malaria parasitological testing were extracted from Kenya DHIS2 between November 2015 and October 2016. DHIS2 public facilities were matched to a geo-coded master facility list to obtain coordinates. Coverage was defined as the geographic distribution of facilities reporting any data by region. Completeness of reporting was defined as the percentage of facilities reporting any data for the whole 12-month period or for 3, 6 and 9 months. **Results:** Public health facilities were 5,933 (59%) of 10,090 extracted. Fifty-nine per Cent of the public facilities did not report any data while 36, 29 and 22% facilities had data reported at least 3, 6 and 9 months, respectively. Only 8% of public facilities had data reported for every month. There were proportionately more hospitals (86%) than health centres (76%) and dispensaries/clinics (30%) reporting. There were significant geographic variations in reporting rates. Counties along the malaria endemic coast had the lowest reporting rate with only 1% of facilities reporting consistently for 12 months.

**Conclusion:** Current coverage and completeness of reporting of malaria parasitological diagnosis across Kenya’s public health system remains poor. The usefulness of routine data to improve our understanding of sub-national heterogeneity across Kenya would require significant improvements to the consistency and coverage of data captured by DHIS2.

## Background

The demand for, and use of, accurate and timely routine malaria data to track the rapidly changing disease epidemiology now forms a major pillar of the latest Global Technical Strategy [1]. The World Health Organization’s T3: Test. Treat. Track. initiative was launched in 2012 [2] and has led to a significant increase across Africa in the numbers of health facilities able to provide malaria parasitological diagnosis, through traditional microscopy or the wide-scale deployment of rapid diagnostic tests [3–5]. Data, however, must be captured in a meaningful way, across all national health service providers every month to provide the granularity required to use this information to track changes in malaria risk with time.

Decades of interest in approaches to improving routine health information data collection has culminated in the latest popular framework, referred to as the District Health Information System version 2 (DHIS2) [6]. DHIS2 provides a platform for all health data, including malaria testing and positivity, to be captured, viewed and analyzed at all levels of the health system from the reporting facility to district and national aggregates. DHIS2 data are increasingly being used to provide national quarterly bulletins of sub-national malaria risk, for example in Uganda, Kenya and Ghana [7–9] and are used by the WHO regional office to compile sub-regional maps of malaria risk [10].

Kenya has a long history of routine health information systems that have allowed for the investigation of the malaria burden [11,12]. In 2011, DHIS2 was launched in Kenya [12,13], the first African country to adopt the online version of the system. In 2016, the Kenyan DHIS2 platform served as the single health reporting system for all allied surveillance systems including malaria commodities, laboratory reporting and the Integrated Disease Surveillance and Response (IDSR) system [14]. Therefore, since 2016, previous fragmented reporting has been avoided under a single-harmonized platform. Here we evaluate the current coverage and completeness of malaria parasitological diagnosis across Kenya’s public health system over a 12-month period between November 2015 and October 2016 at the highest possible resolution by matching reporting coverage to all the country’s public health facilities.

## Methods

### Kenya’s health service providers

In 2010, Kenya adopted a system of decentralized government which included the transfer of management, organization and funding of the health sector to 47 local county governments. The counties are governed by federal health policies with some core functions retained as national functions including immunization and reporting health information.

Formal health services in Kenya are provided by fixed public and private health facilities. Public facilities are maintained by the Government, non-profit Non-Governmental Organizations (NGO) and Faith Based Organizations (FBO) [15]. Service delivery starts at the community level and ends at the national referral hospitals through a hierarchy of healthcare levels [14]. In 2015, among children with fever for whom treatment or advice was sought, 73% received care at public health facilities; this includes 3% who visited faith-based facilities. Private health facilities provided care for 25% of fever cases 3% of whom received care through the retail sector, while 2% received care from community health workers and traditional healers [16]. Fixed service providers are broadly classified into three tiers that support community-level care, increasing in complexity of service provision and staff mixes, from dispensaries/clinics to health centres to hospitals [15,17]. The private sector has grown significantly over the last two decades, however, enumerating and regulating this sector has remained a challenge [18,19]. Unlike the public sector, investments in the capacity of facilities in the private sector to provide any routine data and or better-quality routine data has been lacking [11].

### Mapping service providers

In 2003, the KEMRI – Wellcome Trust Research Programme (KWTRP), in partnership with the Ministry of Health’s (MoH) Division of Malaria Control, initiated an exercise to geo-code a list of formal health service providers developed from multiple MoH department, NGO and FBO listings [20]. This was the first time a map of health service providers had been developed since 1959 [21]. The exercise was repeated using updated information in 2008 [18] and led to the first iteration of MoH’s Kenya master health facility list (KMHFL) in 2009. The KMHFL consists of all health facilities and community units in Kenya with each identified with a unique code with details of administrative location, ownership, type and the services offered. This has been updated at various times, notably during the Service Availability and Readiness Assessment Mapping (SARAM) survey in 2013 [17]. The KMHFL forms the backbone of previous and current health information systems; however, facility coordinates are not available in the public domain and not used by the MoH to map disease data from surveillance systems.

In 2016, we renewed efforts to source additional information on facilities that were no longer operational, those that had changed their level of service provision, checked for duplicates and improved geo-coding using additional online resources including Google Earth [22], Encarta [23], Geonames [24] and OpenStreetMap [25] referred to here as the geo-coded master facility list (MFL).

For the purposes of the present study we have focused on all fixed facilities managed by the Ministry of Health, Local Authorities, FBO and NGO capable of offering general health services to the public. We excluded facilities offering services to a subset population (academic, police, military, prison and company medical services). Other facilities offering specialized care such as HIV Voluntary Counselling and Testing (VCT) centres, specialist disease centres (e.g. tuberculosis, rehabilitation, dental, ophthalmic), family planning clinics, maternity and nursing homes, mobile clinics and blood transfusion centres were also excluded. We have also excluded facilities managed for profit as these remain difficult to map, enumerate and do not enjoy the routine supply of malaria diagnostics.

### Parasitological testing and the DHIS2 platform

Kenya adopted the policy on universal parasitological diagnosis on all cases suspected of malaria in 2010 [26–28] and in 2012, the National Malaria Control Programme (NMCP) embarked on a plan of rolling out rapid diagnostic tests to strengthen the capacity of malaria diagnostic services across the country to all public health facilities without microscopic diagnosis capacity [29].

The DHIS2 routine data platform was adopted in 2010, the same year as universal parasitological diagnosis [26]. The DHIS2 system allows health facilities to report and visualize their monthly data on diseases, commodities and services. A national DHIS2 core team was formed to initiate the implementation of the new system in 2010 and rolled out nationwide between March and September 2011 through cascade training of district health records officers, hospital records officers and District Health Management Team members [12,13]. In 2014, new tools to capture data on suspected malaria cases tested by RDTs and microscopy were developed and incorporated into DHIS2 Kenya platform [14].

### Assembling DHIS2 malaria case reports November 2015 – December 2016

**Figure 1.**
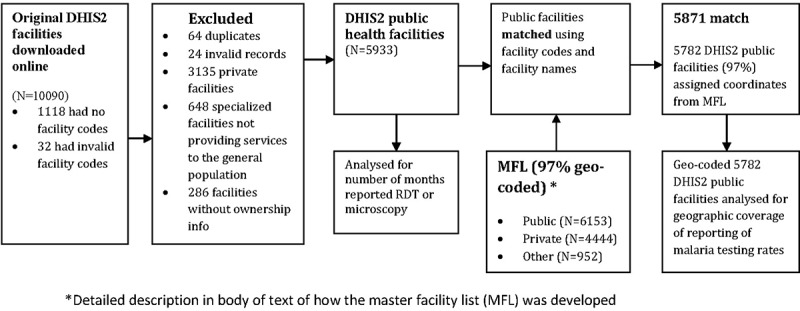
Flow diagram of database processing to generate the data set used in this study.

Monthly records of all malaria indicators covering the period from November 2015 to December 2016 were downloaded in CSV format from the Kenya DHIS2 web portal [30] on 11 April 2017. The dataset included the hierarchy of Kenya DHIS2 organization units of county, sub-county, ward and facility names, facility identification numbers (unique DHIS2 IDs for each facility) and facility codes (5-digit codes used by MoH). To start, the dataset was checked for duplicates and other anomalies which were removed. The cleaned DHIS2 database was linked to the MFL first by facility codes and then by facility names at county level. The matching by facility names was done per county or sub-county due to similar facility names in different counties and sub-counties. Matching facilities were assigned coordinates, facility type and ownership information from the MFL. Through this process, there were 286 facilities whose ownership could not be determined. This process is illustrated in Figure 1.

Data were available for seven malaria indicators and included the number of clinical and confirmed malaria cases; patients tested for malaria by microscopy (<5 and ≥5 years) and RDT and number of positive malaria cases by microscopy and RDT.

We have previously assembled and geo-coded empirical data on *P. falciparum* parasite prevalence (*Pf*PR) from 1980 to 2014 in approximately 3684 unique locations in Kenya [31]. These data were used within a Bayesian hierarchical space-time model implemented through an adapted Stochastic Partial Differential Equations (SPDE) approach using Integrated Nested Laplace Approximations (INLA) for inference [32,33] to develop a 1 × 1km gridded prediction map of parasite prevalence among children aged 2 to 10 years (*Pf*PR_2-10_) projected to the year 2015. The model adjusted for a minimal set of conservative, long-term covariates traditionally used in vector-borne diseases. A detailed methodology used to model this surface is described elsewhere [34].

Data were analysed for the period November 2015 to October 2016 excluding the last two months of 2016 because of a nationwide doctors strike in Kenya. To track testing rates at facilities, data for number tested by microscopy and RDT were analysed across all age groups; data on age (< or > 5 years) of patients tested by RDT was not available. The simplified indicator, therefore, was parasitological tests done (combining microscopy and RDTs). Completeness was evaluated based on the percentage of facilities reporting any data and those facilities reporting data for a total of 3, 6, 9 or 12 months during the period. Consistency of reporting rates was defined here as the number of facilities that had data reported in contiguous months. Data were analysed by facility type (hospital, health centre and dispensary) and malaria risk for facilities offering general health services to the public and for only those that had reported at least once for any indicator on the DHIS2 system. The geo-coded facilities were plotted on a map of Kenya counties to assess geographic variation in completeness of reporting.

## Results

**Figure 2.**
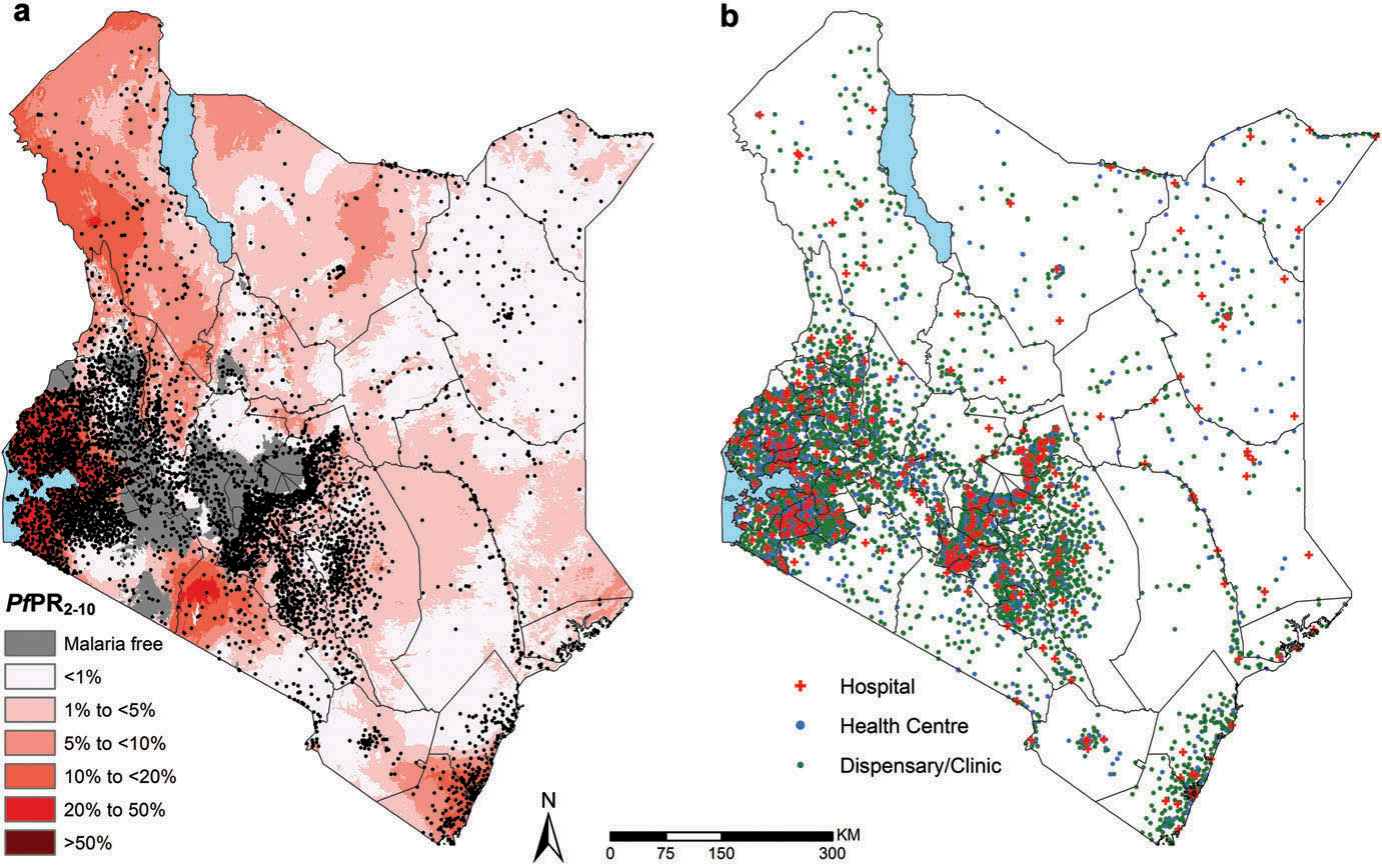
Distribution of the 5782 geocoded public health facilities in DHIS2 with coordinates out of the 5933 public facilities. (a) – Location of all facility types overlaid on a malaria endemicity map of *Plasmodium falciparum* prevalence in 2015 among children 2–10 years of age (*Pf*PR_2-10_) in Kenya at 1 × 1 km spatial resolution [31] (b) Map showing the location of facilities categorized by facility types.

The dataset extracted from the Kenya DHIS2 web portal had 10,090 facilities. Facilities that did not have facility codes were 1,118 (11%) while 32 (0.3%) facilities had invalid facility codes. Excluded facilities were 64 duplicates, 13 facilities coded as closed and inactive, and an additional 11 invalid records. A total of 3871 (38%) non-public facilities and 286 (3%) facilities for which ownership could not be determined were excluded. The final dataset had 5,933 public health facilities of which 7% were hospitals, 17% health centres and 76% either clinics or dispensaries, all without coordinates (Table 1). Facilities that were successfully matched to facilities in the MFL were 5,871 (99%) where 5,782 (97%) of the DHIS2 facilities were assigned coordinates (Figure 2).

**Table 1.** A Description of public health facilities included in this study.

Type of facility	Total number of Public facilities (N = 5933)
Dispensaries & clinics	4,527 (76%)
Health Centres	1,010 (17%)
Hospitals	396 (7%)
**Number of facilities with Geocodes**	
Facilities with coordinates	5782 (97%)
Facilities without coordinates	151 (3%)

### Completeness of data on testing rates reported in public health facilities

In 2015–16, 59% (n = 3487) of public health facilities did not report any malaria diagnostic test results for any single month (Table 2). 36%, 29% and 22% facilities had data on testing rates reported in at least 3, 6 and 9 months respectively in the 12-month period under review. Among the 2,446 facilities (41%) with some data reported on malaria testing, 88%, 71% and 52% had at least 3, 6 and 9 months of data reported in the DHIS2 platform, respectively. Only 8% of public health facilities reported malaria testing rates every month between November 2015 and October 2016.

**Table 2.** Reporting patterns for malaria testing rates from Public health facilities based on reporting rates available from DHIS2 between November 2015 and October 2016.

Period	Public Health Facilities in DHIS2 (N = 5933)	
	Number of facilities with contiguous months of data on malaria BS or RDT reported n (%)	Number of facilities with non-contiguous months of data on malaria BS or RDT reported n (%)
Any month	NA	2,446 (41%)
At least 3 months	1997 (34%)	2,162 (36%)
At least 6 months	1,306 (22%)	1,746 (29%)
At least 9 months	771 (13%)	1,284 (22%)
For 12 months	452 (8%)	452 (8%)

**Figure 3.**
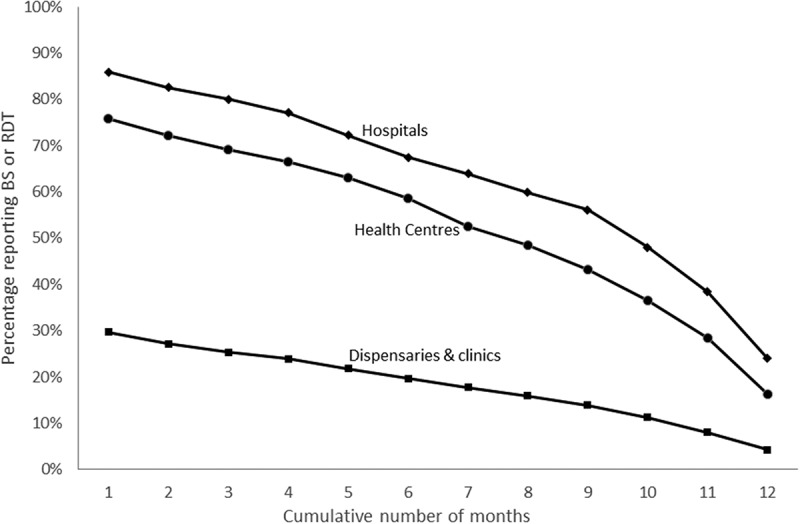
Number of facilities with data on BS or RDT done reported by month. Showing the scale of completeness of reporting by level of facility in DHIS2 by month between November 2015 and October 2016.

We analysed completeness of reporting of malaria parasitological diagnosis by level of facility – hospitals, health centres and dispensaries/clinics. There was a stable decline in completeness of reporting rates across all levels with increasing number of months of data expected (Figure 3). A larger proportion of hospitals (86%; n = 340/396) were likely to have data on testing rates reported in any month compared to health centres (76%; n = 765/1010) and dispensaries/clinics 30% (n = 1341/4527). The rate of consistency in reporting (data on malaria parasitological diagnosis reported in all 12 months) was also highest in hospitals at 24% compared with 16% of health centres and 4% of clinics/dispensaries.

We evaluated the consistency of reporting patterns across facilities using the metric of the number of contiguous months of malaria test data reported on the DHIS2 platform (Table 2). There were only 34% (n = 1997) of public DHIS2 facilities with data reported in any 3 consecutive months while 36% (n = 2162) reported data in any 3 months (Table 2). Facilities that reported data in 6 and 9 consecutive months were 1,306 (22%) and 771 (13%) respectively while 1,746 (29%) and 1,284 (22%) reported in any 6 or 9 months, respectively.

### Spatial analysis of trends in testing rates

**Figure 4.**
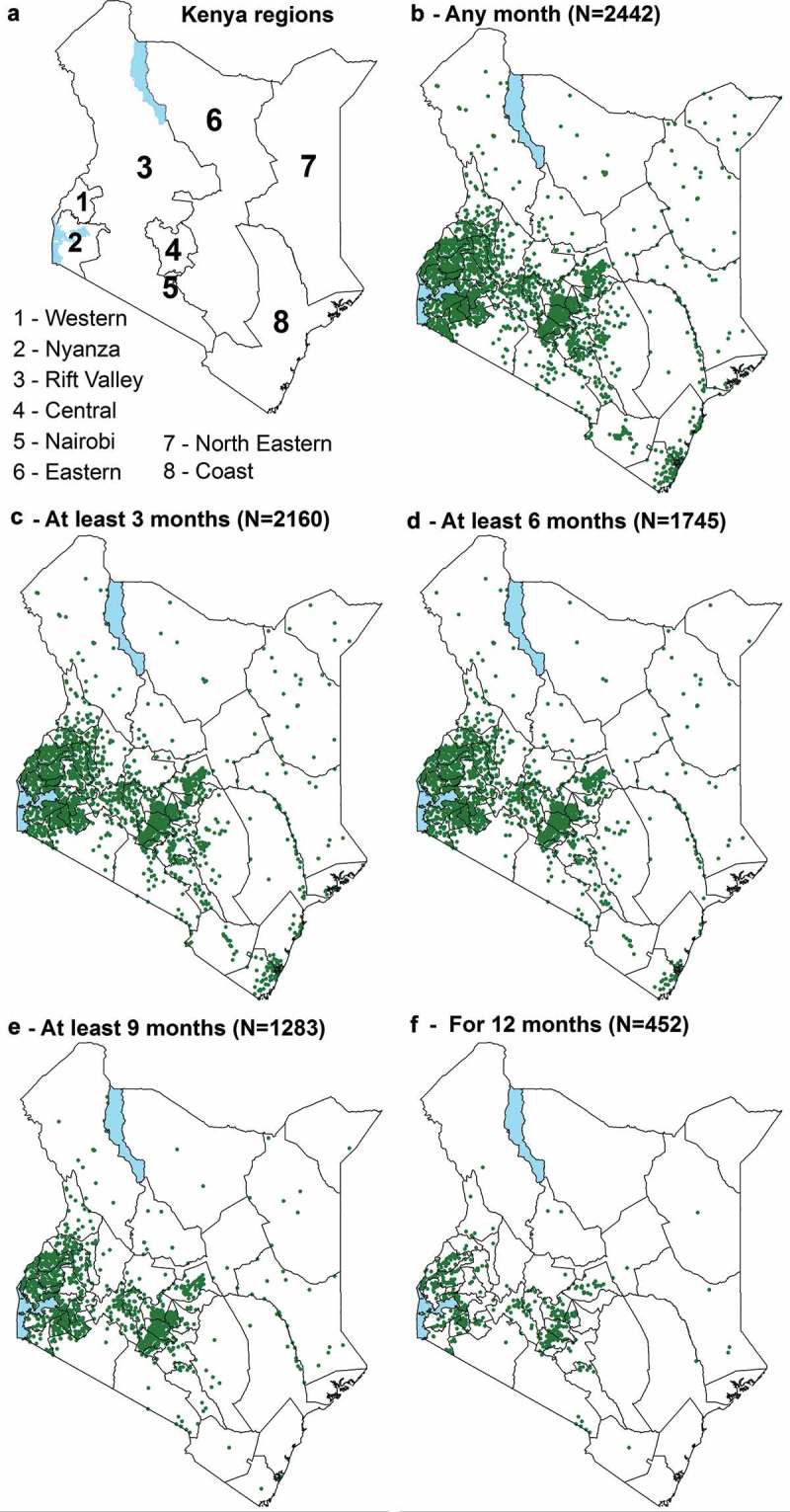
Distribution of public DHIS2 facilities reporting BS or RDT between November 2015 and October 2016 plotted on a map of counties. (a) – Regions of Kenya. (b) – 2442 geocoded public health facilities with slides or RDT reported in DHIS2 of 2446 in any month. (c) – 2160 geocoded public health facilities with slides or RDT reported in DHIS2 for at least 3 months. (d) – 1745 geocoded public health facilities with slides or RDT reported in DHIS2 for at least 6 months. (e) – 1283 geocoded public health facilities with slides or RDT reported in DHIS2 for at least 9 months. (f) – 452 geocoded public health facilities with slides or RDT reported in DHIS2 for 12 months. This is restricted to those facilities with coordinates.

Figure 4(a–f) shows reporting rates and the completeness of reporting by different geographic regions across Kenya for the 5782 public health facilities with geo-coordinates. Coverage rates varied by region with uneven reporting rates nationally. Counties along the coast of Kenya, which are some of the counties with the highest malaria burden [35], had the lowest reporting and completeness rates recorded. The highest coverage was in counties in Western and Nyanza regions. Similar high coverage was observed in the Central Kenyan region which has almost no malaria [31,35].

## Discussion

There is increasing recognition of the importance of robust routine data in tracking progress in malaria control which has presented an opportunity to strengthen national routine data and surveillance reporting systems. In Kenya, reporting across multiple data platforms has been harmonized into a unified DHIS2 platform since 2016. In this study, we sought to evaluate the coverage and completeness of reporting of malaria parasitological diagnosis in the public health system through DHIS2. Such an examination of trends in routine surveillance data on, parasitological diagnosis, cases and deaths recorded is a natural starting point for assessing change and for use in assessments of seasonal patterns of malaria. To exploit the utility of these data for such purposes would require data in every month and year as well as a reliable estimate of the total number of facilities and their distribution. Our assessment shows that between November 2015 and December 2016, a period spanning 12 months, reporting rates remained poor, at only 41% of known public health facilities in Kenya reporting any data onto the DHIS2 platform. Of those public facilities that reported any data, less than 10% had consistent reporting rates, that is, they had data reported in over the 12 months. DHIS2 has been used previously to capture malaria-related information and has helped improve countries’ understanding of the links between malaria disease burden, use of rapid diagnostic tests, and administration of anti-malarial drugs administered [36,37]. However, similar evaluations of the coverage and completeness of data captured in national DHIS 2.0 systems are few [11–13,38,39]. There have also been several attempts to use routine data in model-based geostatistics using data from Namibia [40], Afghanistan [41] and in Madagascar [42] and these have the potential to form part of new strategies for malaria risk mapping in future [41,43].

Routine data provide an avenue through which our understanding of sub-national heterogeneity can be enhanced. However, in this study, in spite of the fact that geographic coverage was generally low, there were noticeable differences in reporting rates observed across different geographies with counties along the Coast of Kenya having much lower complete coverage rates. Only 1% of facilities along the coast had data reported in all 12 months compared with 13% in Western Kenya and 12% in Central Kenya. Data collected within the DHIS2 platform has huge potential and can be useful in planning and better targeting of interventions, however for this to be realised, nationally reliable and representative coverage is necessary.

Private facilities were not included in this analysis. In Kenya, the private sector constitutes slightly less than half of the facilities in the Kenyan health system however, we remain ignorant of the coverage of routine reporting in private facilities as these typically are harder to geo-locate; we couldn’t position nearly a quarter of the private facilities in our final database. Additionally, services in these facilities are not readily available to the public and such facilities are also not well regulated and therefore the quality of services offered is more difficult to assess. We therefore opted to exclude these facilities. Additionally, activities in these facilities typically do not rely on government or donor-funding hence the need for and use of standardized data collection tools necessary for monitoring and evaluation characteristic of increased demand for data seen in much of the public sector is non-existent. Hence the private sector remains characterized by poorer reporting rates [11,44].

It was not possible within the confines of this study to evaluate the quality of the data recorded [45]. Nonetheless, even if the quality of these data were good, coverage remains poor. It was also not feasible to explore the reasons underlying the missing data such as the availability of resources at facility with which to complete indicators nor was it possible to evaluate the possible role of systemic problems with data recording nor the extent to which these results reflect a problem with the supply chain of diagnostics. This seems unlikely given previous work on the diagnostic supply chains [46,47]. We restricted the analysis to a review of testing rates because for some of the other indicators we were unable to make a distinction between instances where no data were reported (true missing) from those instances where there were no positive cases because of how the data were recorded in the DHIS2 platform (zero vs blanks for the number positive) hence these data were not analyzed.

## Conclusion

The T3 approach to malaria case management and surveillance is a significant transition from traditional presumptive diagnosis and poor health information systems in Africa. The current focus is on ensuring adequate testing rates and rationalizing treatment to those who test positive. Far less is understood about the coverage and quality of the third T, tracking [48–51]. This study gives a sense of the usefulness of the current state of routine data in providing the granularity of data needed to begin to redesign and tailor interventions to those areas in greatest need. However, results from this study show that coverage rates, completeness and the consistency of tracking testing rates remain extremely low and importantly was poorer in regions where malaria is endemic. Such localized information is essential for malaria programs to be more responsive to local needs. By focusing on improving the coverage and regularity of data collected through DHIS2, countries can begin to more accurately track progress using high quality, timely routine health data and information and make strides towards the new SDGs.

## Paper context

Kenya launched DHIS2 in 2011 to be the main health management information system. Kenyan health facilities began reporting routine malaria parasitological testing data into DHIS2 in January 2015. This study will interrogate the national spatial and temporal reporting patterns on malaria parasitological testing data in Kenya DHIS2 between 2015 and 2016 at facility level and will provide DHIS2 stakeholders and decision makers a clear picture of where reporting improvements need to be focused.
